# Supplementary material for: High endogenous CCL2 expression promotes the aggressive phenotype of human inflammatory breast cancer
Source: Nat Commun. 2021 Nov 25;12:6889. doi: 10.1038/s41467-021-27108-8 (PMC8617270; doi:10.1038/s41467-021-27108-8)
Supplement: Supplementary file 1 — Supplementary Information [file 41467_2021_27108_MOESM1_ESM.pdf]

Supplemental Figure 1

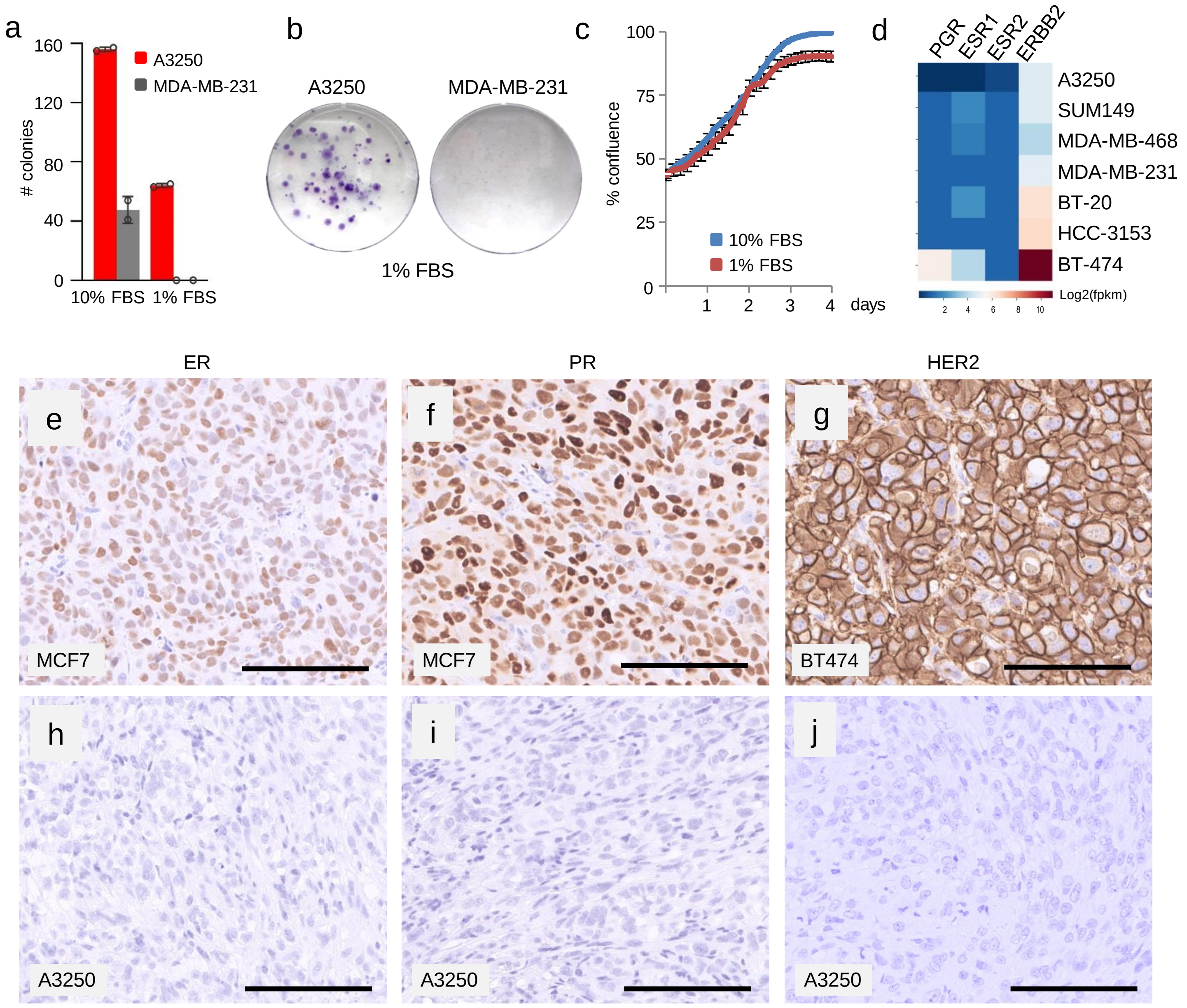

Supplemental Figure 1. Growth characteristics of A3250 tumor cells in vitro and expression of hormone receptors. (a, b) Colony formation in high or low serum as indicated, at seeding density of 52 cells/cm^2. Quantification (a) and representative images (b). Data are presented as mean values ± SD. *n*=3 biologically independent samples per cell line per condition. Two independent experiments were performed. (c) Kinetics of A3250 tumor cell proliferation in vitro assessed by IncuCyte live-cell analysis system. *n*=3 biologically independent samples per condition. Data are presented as mean values ± SD Two independent experiments were performed. (d) Expression of *ER (ESR), PR (PGR)*, and *HER2 (ERBB2)* determined by mRNA-Seq in A3250 cells in comparison to other breast cancer cell lines. (e-j) Immunostaining for ER (e, h), PR (f, i) and HER2 (g, j) of tumors as indicated. Images are representative of two independent experiments. *n*=3 biologically independent mice. Scale bars: 100 µm. FPKM = Fragments Per Kilobase of exon per Million mapped reads. Source data are provided as a Source Data file.

Supplemental Figure 2

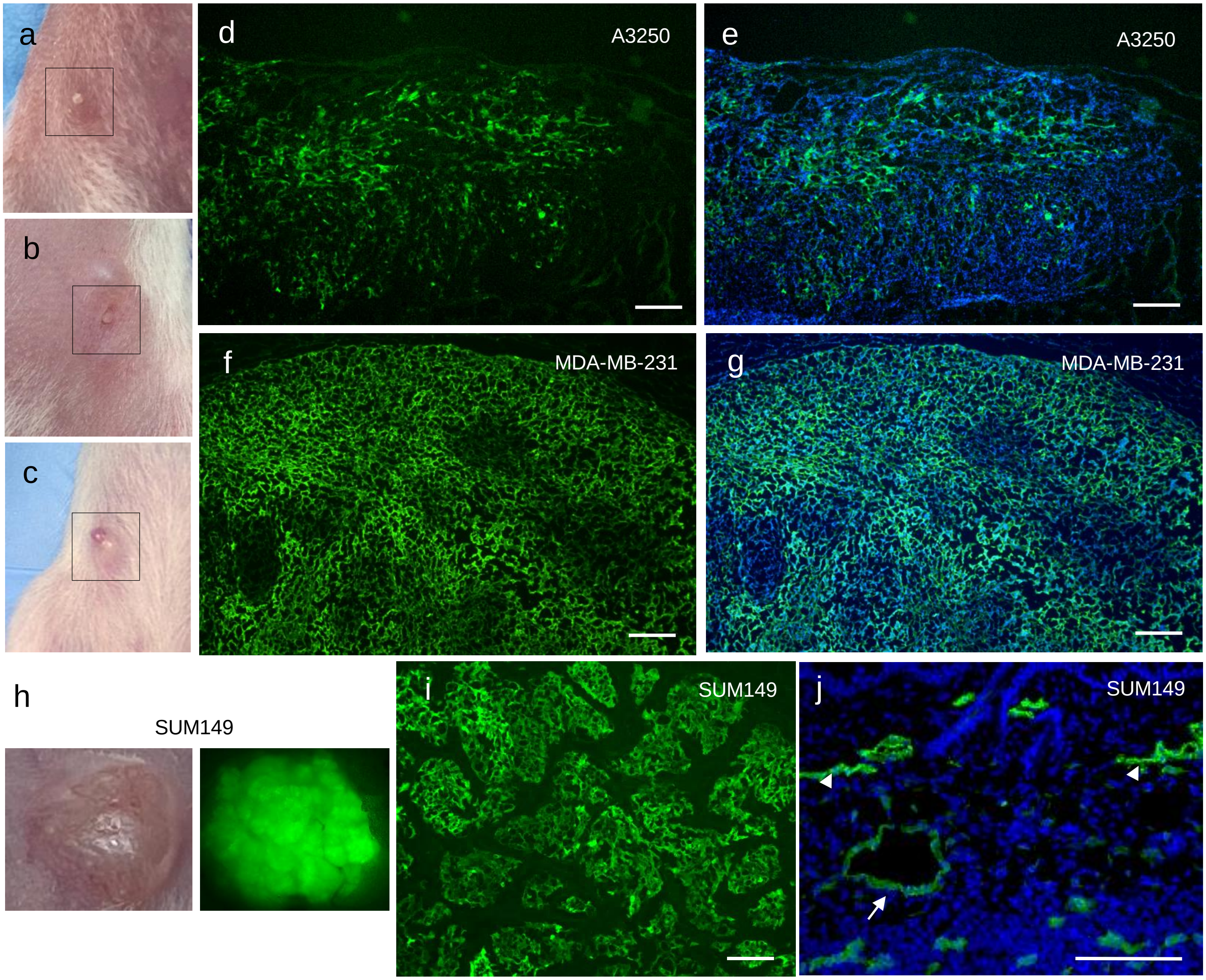

Supplemental Figure 2. In vivo growth characteristics of A3250, SUM149 and MDA-MB-231 tumors. (a-c) Nipple abnormalities associated with A3250 tumor formation. Normal mouse nipple (a), nipple inversion (b) and protrusion (c). (d-g) Tumor cells visualized by GFP (green) in 6 mm sections of tumors as indicated. Note diffuse growth of A3250 tumors. (h) Representative bright field image and fluorescence stereo-microscopy of a SUM149 tumor ex vivo at four weeks (GFP, green). (i) Section (6 mm) of a SUM149 tumor at four weeks (GFP, green). (j) Immunofluorescent staining of skin adjacent to a SUM149 for lymphatic marker LYVE-1 (green) at four weeks post-injection. Arrow points to a dilated lymphatic vessel in the dermis, note absence of tumor emboli in lymphatics. Arrowheads point to normal, not dilated lymphatics. Images are representative of two independent experiments. *n*=5 biologically independent mice. DAPI, blue. Scale bars: d-g, 200 μm; i-m 100 μm.

Supplemental Figure 3

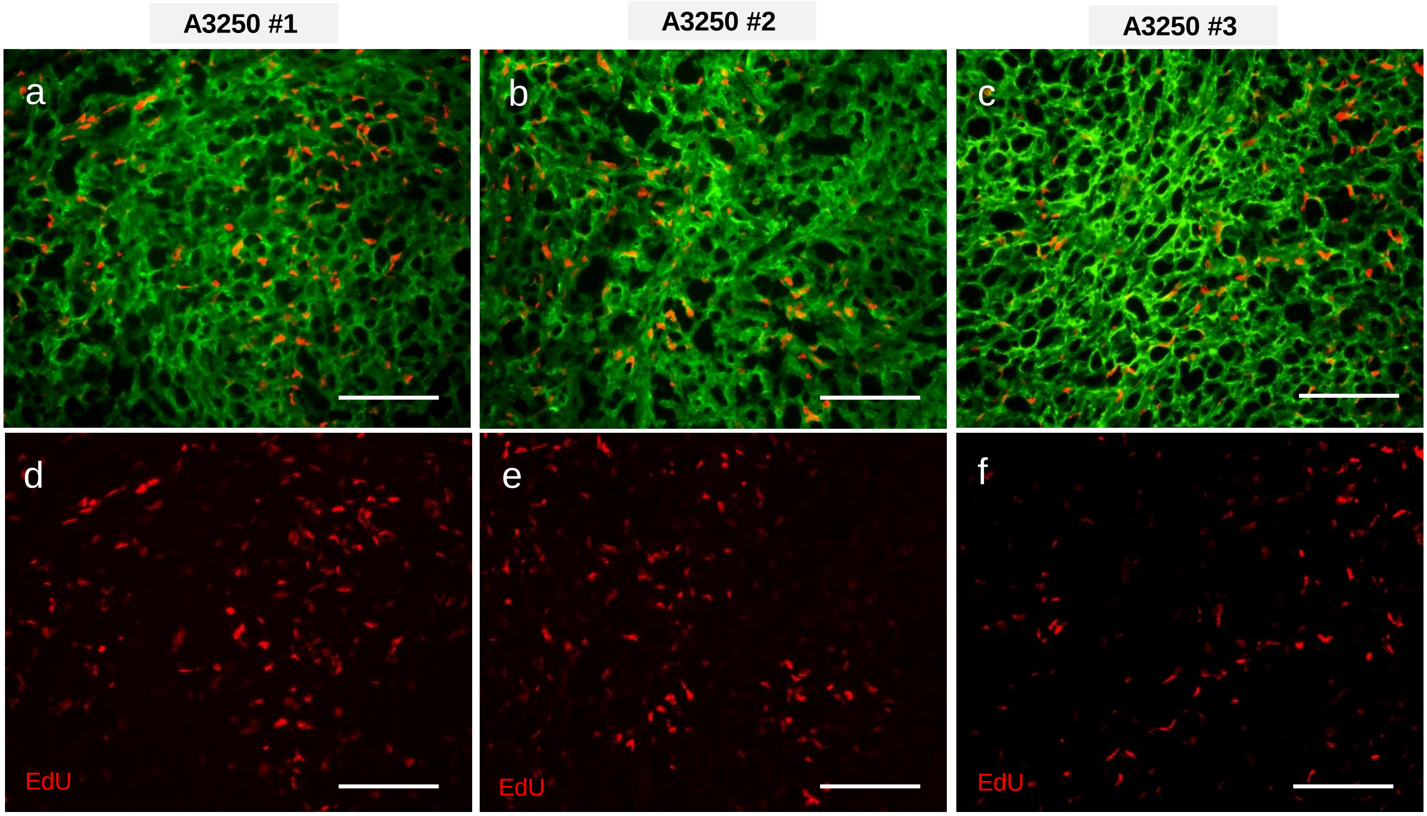

Supplemental Figure 3. A3250 tumor proliferation. (a-f) Representative images of three A3250 tumors stained for EdU (red) and GFP (green). Images are representative of two independent experiments. Scale bars: 100 µm.

Supplemental Figure 4

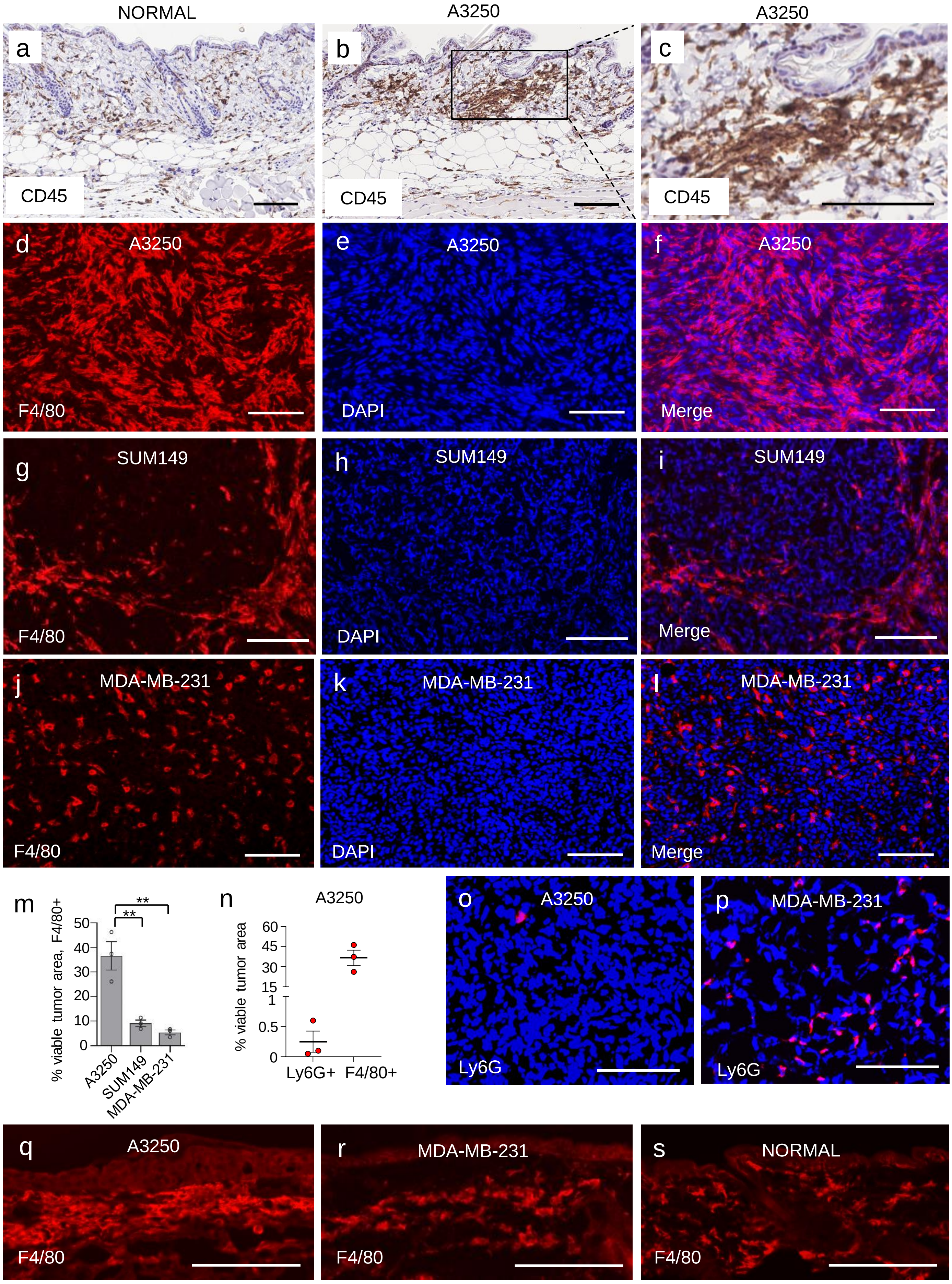

Supplemental Figure 4. Macrophages in A3250, SUM149 and MDA-MB-231 tumors. (a-c) CD45 immunostaining (brown) of the skin from normal, tumor-naïve mouse (A), and skin adjacent to a A3250 tumor (b, c). (d-l) Immunofluorescent staining for macrophage marker F4/80 (red) in tumors as indicated. (m) Quantification of F4/80+ cells in tumors as indicated based on immunostaining (n=3 biologically independent samples per cell line). One-way ANOVA yielded p=0.0014 and unpaired, two-tailed t-tests yielded p values of 0.01 (A3250 vs. SUM149) and 0.0062 (A3250 vs. MDA-MB-231). (n) Quantification of F4/80+ and Ly6G+ cells in A3250 tumors based on immunostaining (n=3 biologically independent samples). (o, p) Immunostaining for Ly6G in tumors as indicated. (q-s) Skin overlaying A3250 or MDA-MB-231 tumors and skin of normal SCID mice immunostained for F4/80. Tumors are four-weeks old and control mice are age-matched. Images are representative of two independent experiments. *n*=3 biologically independent mice. DAPI, blue. Scale bars: 100 μm. m, n: data presented as mean values ± SEM. **p<0.01. Source data are provided as a Source Data file.

Supplemental Figure 5

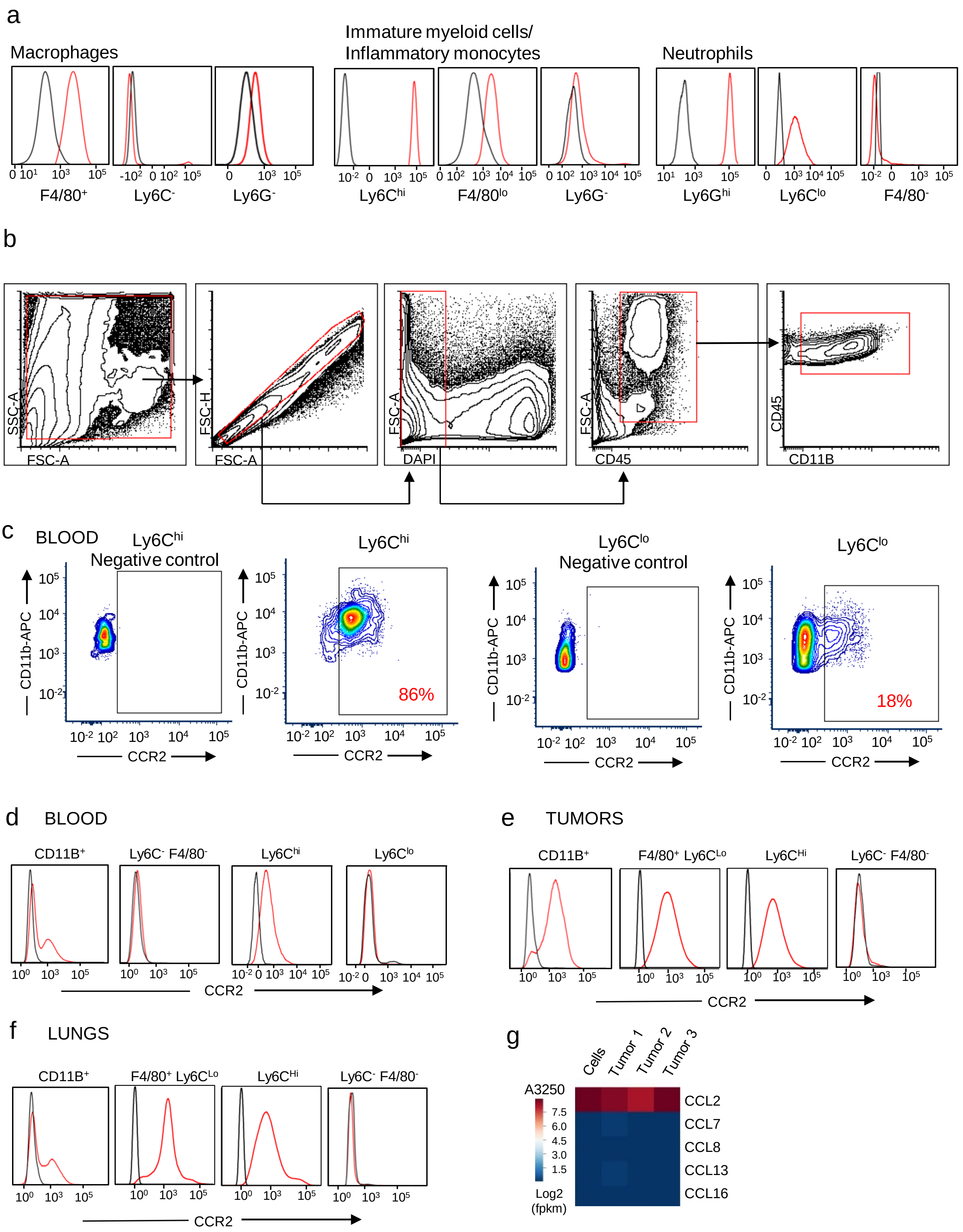

Supplemental Figure 5. Characterization of inflammatory cells in A3250 tumor model. (a) Flow cytometry histograms showing surface marker profile for the main subsets of inflammatory cells seen in four weeks old A3250 tumors. (b) Gating strategy to identify viable CD45+CD11b+ myeloid cells, presented in Fig. 5a. (c) Representative FACS plots showing CCR2 expression on Ly6Chigh and Ly6Clow monocytes in blood of mice bearing A3250 tumors. (d-f) FACS analysis of CCR2 expression on relevant CD11b+ cell subsets in blood (d), primary tumors (e) and lungs (f) from A3250 tumor bearing mice. (g) Heatmap showing expression of human CCR2 ligands in A3250 cells and tumors (n=3) determined by mRNA-Seq. FPKM = Fragments Per Kilobase of exon per Million mapped reads.

Supplemental Figure 6

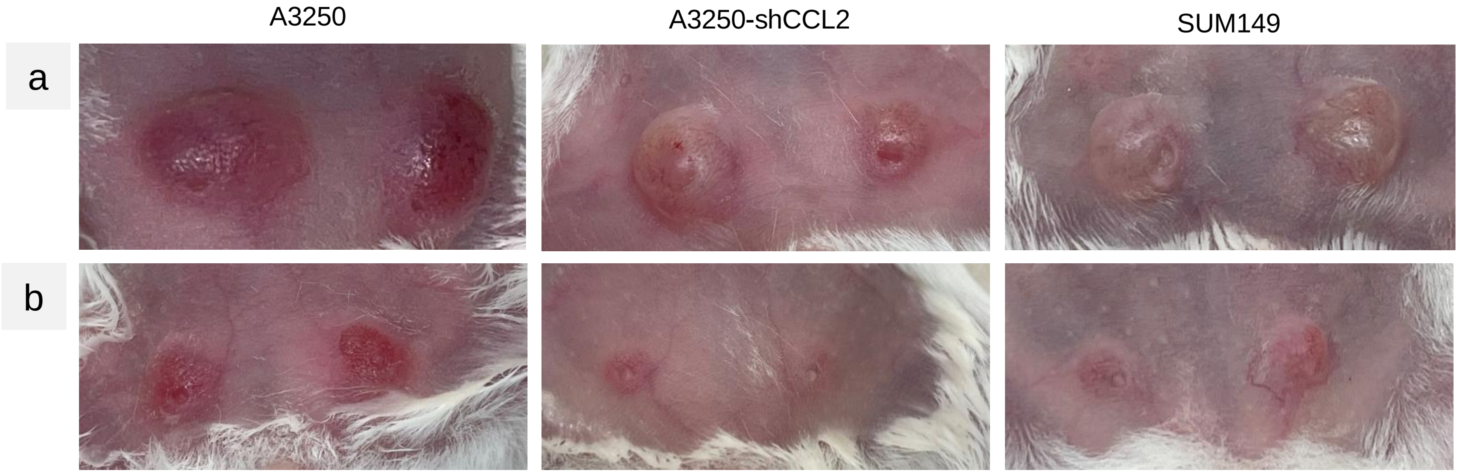

c

	Redness	Nipple changes	Peau d'orange
A3250 control	9/10	7/10	9/10
A3250-shCCL2	0/5	2/5	3/5

Supplemental Figure 6. Erythema and gross appearance of A3250 tumors. (a, b) Representative images of erythema associated with large tumors (a) and small tumors (b) in mice as indicated. (c) Incidence of clinical symptoms characteristic of IBC in A3250 and A3250-shCCL2 tumors. Images are representative of two independent experiments. *n*=10 mice in control, and *n*=5 mice in shCCL2 group.

Supplemental Figure 7

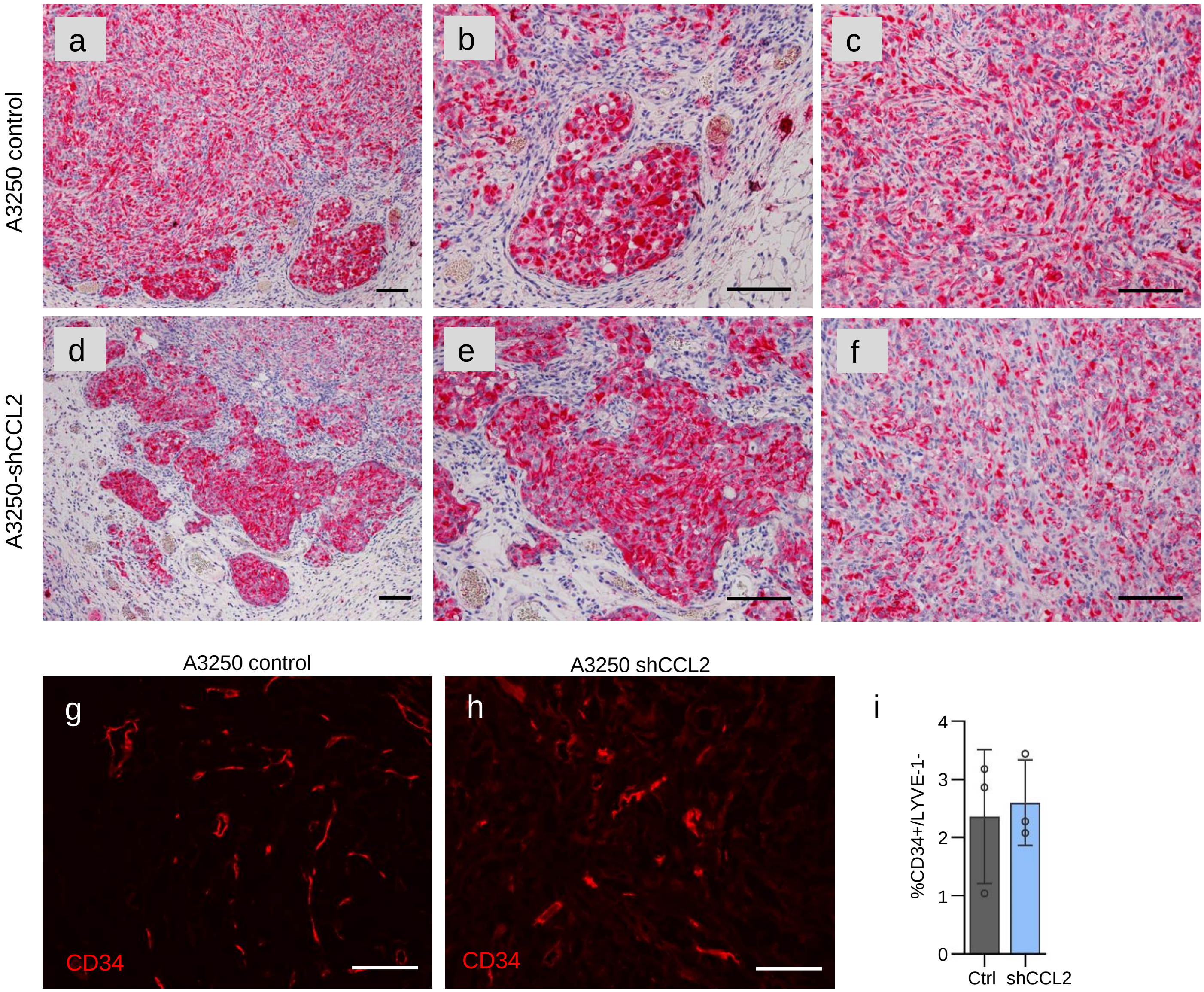

Supplemental Figure 7. Invasion and angiogenesis in A3250 control and shCCL2 tumors. (a-f) IHC for pan-cytokeratin on tumors as indicated. (a-c) Representative images of A3250 control tumor. Invasive margin at low (a) and high magnification (b) and corresponding primary tumor mass (c). (d-f) Representative images of A3250-shCCL2 tumor. Invasive margin at low (d) and high magnification (e) and corresponding primary tumor mass (f). (g, h) Immunofluorescent staining for CD34 to visualize blood vessels in tumor sections as indicated. (i) Quantification of CD34+/LYVE-1- blood vessels in A3250 control and shCCL2 KD tumors. *n*=3 biologically independent samples per cell line. Images are representative of two independent experiments. Data is presented as mean values ± SD. Scale bars: 100 µm.

Supplemental Figure 8

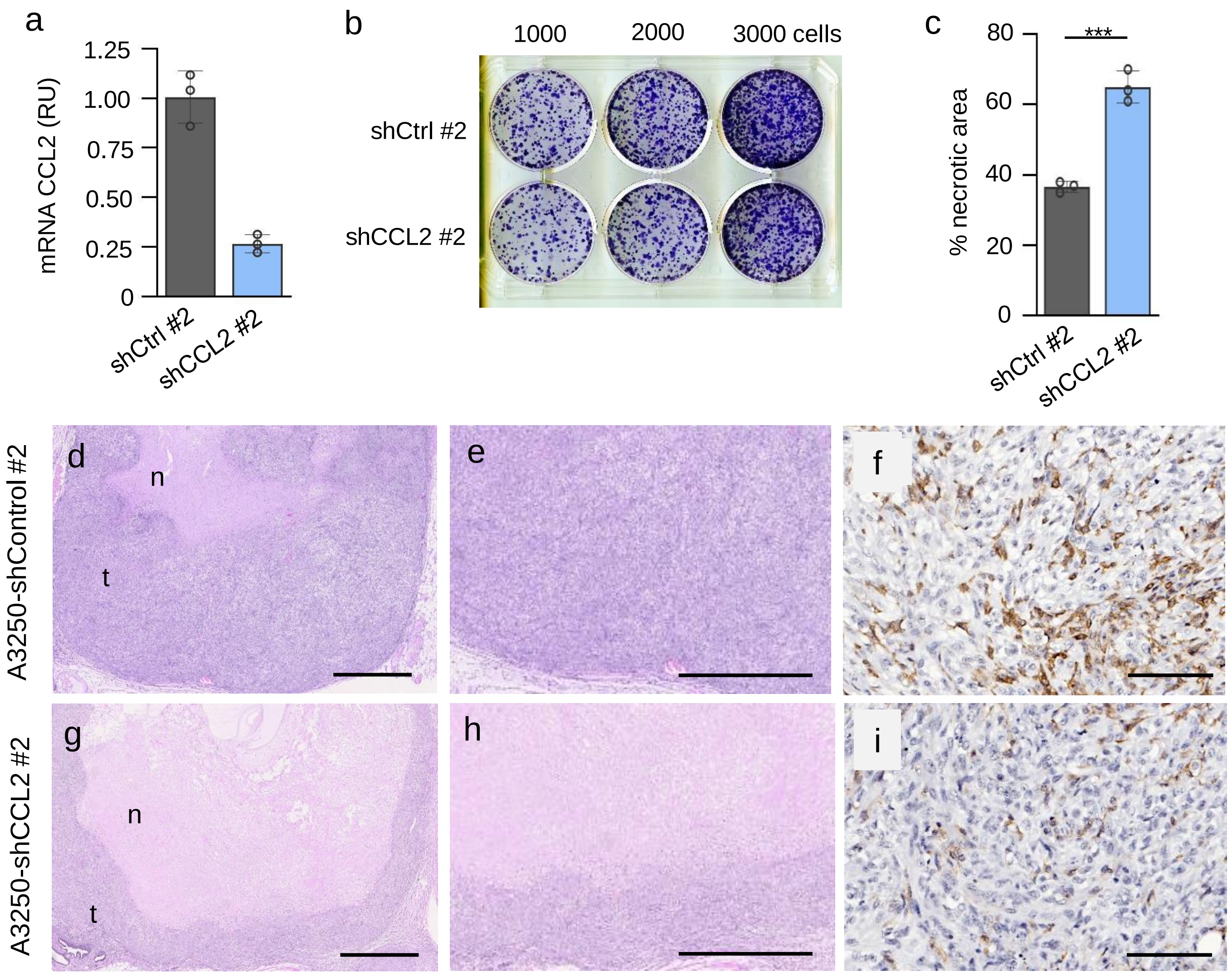

Supplemental Figure 8. Growth characteristics of A3250-shCCL2 #2 tumor cells in vitro and in vivo. (a) qPCR for CCL2 expression in A3250-shControl#2 cells in vitro compared to A3250-shCCL2#2 cells. *n*=3 independent experiments per cell line. Data are presented as mean values ± SD. (b) Representative image of colony-formation at various seeding densities. (c) Quantification of necrotic tumor areas based on H&E staining (*n*=3 biologically independent tumors per cell line). Data are presented as mean values ± SEM. *p*=0.0005, determined through an unpaired two-tailed t-test. (d-i) Representative images of A3250-shControl#2 (d, e) and A3250-shCCL2#2 (g, h) tumors stained for H&E, showing necrotic (n) and viable tumor (t) areas. (f, i) IHC staining for CD45 of A3250-shControl (f) and A3250-shCCL2 (i) tumor sections. Images are representative of one experiment. *n*=3 biologically independent mice. Scale bars: d, g, 1 mm; e, h: 500 μM; f, i: 100 μM. ***p<0.001

Supplemental Figure 9

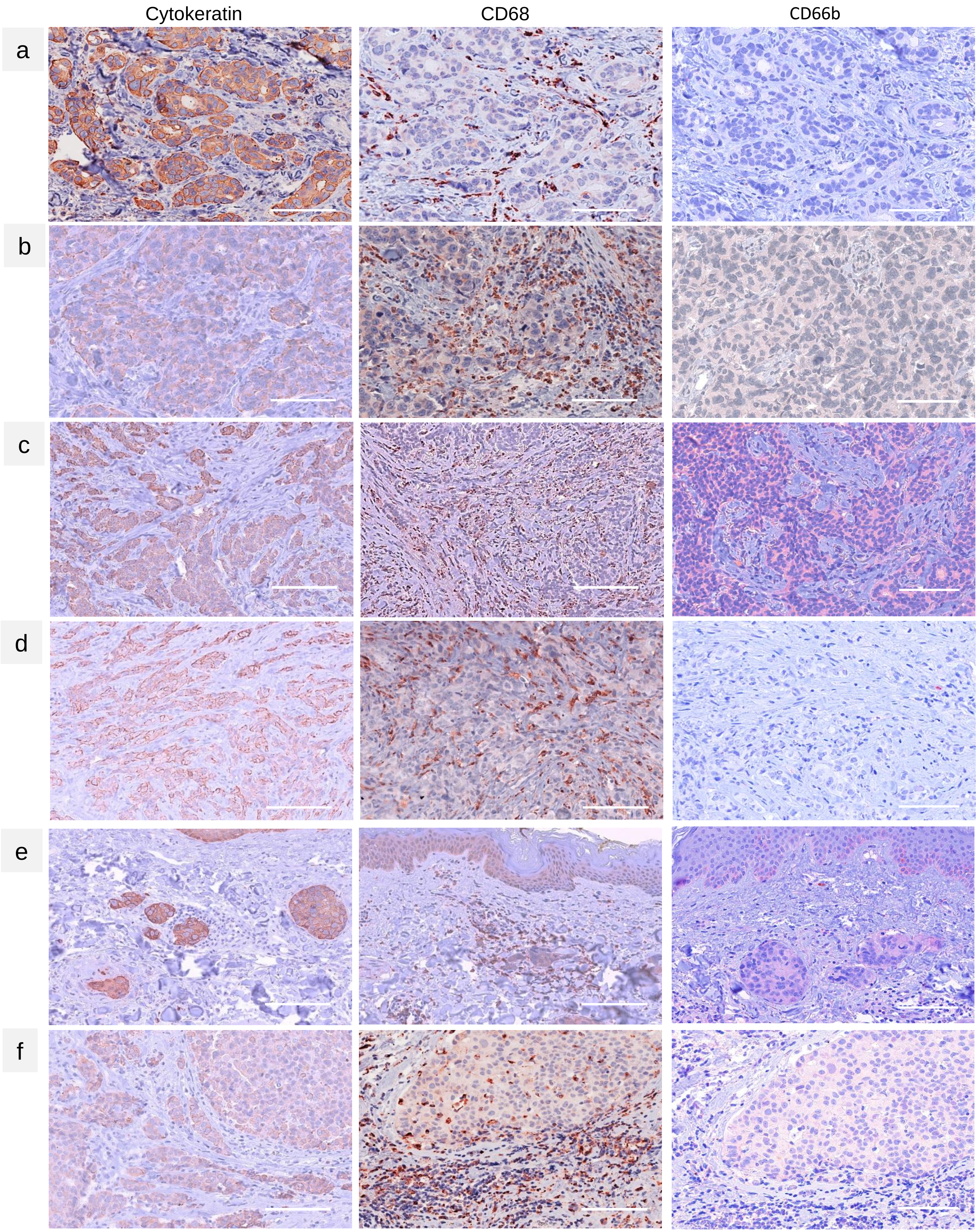

Supplemental Figure 9. Macrophages and neutrophils in human IBC. (a-f) Representative images of six human IBCs immunostained with antibodies against cytokeratin, CD68 (macrophages) and CD66b (neutrophils). Each panel shows serial sections of one tumor. *n*=6 tumors. Note prominent presence of macrophages and absence of neutrophils. Scale bars: 100 µm.

Suppl. Table1: Shot Tandem Repeat (STR) DNA Profile Report for A3250 cells.

ATCC cell line authentication is based on 17 STR markers plus amelogenin for gender determination, which creates a unique genetic signature. Values shown in columns B and C represent allele scores. Allele score is the number of tandem repeats (STR) present in each allele of the given genetic locus. Column B shows number of tandem repeats for allele 1, and column C shows number of tandem repeats for allele 2 for the given locus. One allele score is shown in cases where only one allele was present. The STR profile of the A3250 cell line is compared to profiles of other cell lines in the reference database and was determined to be unique.

Locus	Allele score(s)	
D3S1358	15	18
TH01	6	7
D21S11	28	30
D18S51	12	16
Penta_E	7	12
D5S818	11	
D13S317	10	13
D7S820	11	
D16S539	11	12
CSF1PO	11	
Penta_D	9	14
vWA	17	19
D8S1179	11	14
TPOX	8	9
FGA	21	24
D19S433	13	
D2S1338	20	24
Amelogenin	X chromosome	

Suppl. Table 2: ER, PR and ERBB2 mRNA expression levels (FPKM) in A3250 and other breast cancer cell lines by mRNA-Seq. Comparison to TNBC cell lines and BT474 Her2+ breast cancer cell line. For A3250 tumors, A3250 cells, SUM149 and MDA-MB-231, average values are shown (n=3). Data for other cell lines were from the GSE27003 dataset (n=1).

Symbol	Gene Name	A3250 tumors	A3250 cells	SUM149	MDA-MB-231	MDA-MB-468	BT20	BT474
ESR1	estrogen receptor 1	0	0	0	0	1.1	2.43	12.54
ESR2	estrogen receptor 2	0	0	0	0	0	0	0
PGR	progesterone receptor	0	0	0	0	0	0	55.73
ERBB2	erb-b2 receptor tyrosine kinase 2	27.98	17.47	7.88	6.71	13.22	75.23	1939.25

Suppl. Table 3: Quantification of EdU+ proliferating tumor cells in A3250 tumors.
EdU signal was quantified in GFP+ tumor areas. EdU pulse was 6 hr.

CONTROL

Sample	Total GFP+ area (mm2)	EdU+ area (mm2)	EdU+ area/GFP+ tumor area	Edu+ cells/GFP+ area	Edu+ cells/mm2 area (per 6 hr)
Tumor 1	0.629	0.058553	0.093	330	525
Tumor 2	0.691	0.047186	0.068	266	385
Tumor 3	0.791	0.065315	0.083	368	465
**Average**	**0.704**	**0.058018**	**0.081**	**321**	**458**
STDDEV	0.082	0.009161	0.013	51.5	70.2

CCL2 KD

Sample	Total GFP+ area (mm2)	EdU+ area (mm2)	EdU+ area/GFP+ tumor area	Edu+ cells/GFP+ area	Edu+ cells/mm2 area (per 6 hr)
Tumor 1	0.662	0.006	0.009	36	55
Tumor 2	0.514	0.007	0.014	40	78
Tumor 3	0.667	0.012	0.018	69	103
**Average**	**0.614**	**0.008**	**0.014**	**48**	**78**
STDDEV	0.087	0.003	0.005	18	24

***p*-value: 0.0009** - determined through unpaired, two-tailed t-test
Control vs. CCL2 KD

Suppl. Table 4: Chemokines expressed by A3250 cells compared to other breast cancer cell lines by mRNA-Seq.

Values shown are in FPKM. The criteria for clasifying a gene as expressed is FPKM≥1 in all samples analyzed per condition.

For experiments with *n*=3, average values are shown. A3250, SUM149 and MDA-MB-231 were sequenced in-house. Data for IBC3 was from the GSM3145605 dataset, HCC3153 and BT-20 were from the GSE27003 dataset.
FPKM values were rounded to the nearest whole number.

Gene	A3250, Exp 1	A3250, Exp 2	SUM149	IBC3	MDA-MB-231	HCC3153	BT-20
CCL2	467	441	0	5	0	1	2
CXCL5	351	997	12	0	0	0	0
CCL20	17	16	23	0	0	3	1
CXCL2	13	67	10	1	0	6	0
CXCL3	1	6	23	1	0	4	0
CXCL16	4	6	46	7	2	1	46
CXCL1	11	101	225	1	0	27	0
	n=1	n=3	n=3	n=1	n=3	n=1	n=1

Suppl. Table 5: Olink proteomic analysis of select chemokines in A3250, SUM149 and MDA-MB-231 cells

Protein concentration determined in cell superntants is shown in NPX units (Normalized Protein Expression). NPX is an arbitrary unit on a Log2 scale.

	**A3250**		**MDA-MB-231**		**SUM149**	
	24 hr	48 hr	24 hr	48 hr	24 hr	48 hr
CXCL5	12.38	12.16	0.93	1.44	9.27	10.41
CCL20	7.09	9.43	1.42	2.27	7.33	8.61
CXCL1	12.89	12.96	8.45	9.02	12.30	12.68
CCL2	11.39	11.34	1.21	1.90	0.27	0.32

Suppl. Table 6: Expression of human CCR2 ligands by A3250 tumors and cells in vitro and in vivo by mRNA-Seq.
Values shown are FPKM. For A3250 tumors and cells where *n*=3, average value is shown.

	A3250, in vitro	A3250, in vitro	shControl, in vitro	shCCL2, in vitro	A3250, tumors	shControl, tumors	shCCL2, tumors
CCL2	467.22	441.16	261.60	75.04	388.32	220.19	86.1
CCL7	0.07	0.03	0.06	0.08	0.09	0.07	0.1
CCL8	0.04	0.02	0.04	0.05	0.06	0.04	0.06
CCL13	0.06	0.03	0.05	0.06	0.09	0.06	0.09
CCL16	0.02	0.01	0.02	0.03	0.03	0.02	0.03
	Exp1	Exp 2	Exp1	Exp1	Exp1	Exp1	Exp1
	n=1	n=3	n=1	n=1	n=3	n=3	n=3

Suppl. Table 7: Expression of CCR2 in A3250 cells and tumors by mRNA-Seq. Values shown are FPKM. For A3250 tumors, average value is shown (*n*=3). For in vitro, *n*=1.

	**A3250, in vitro**	**shControl, in vitro**	**shCCL2, in vitro**	**A3250, tumors**	**shControl, tumors**	**shCCL2, tumors**
human CCR2	0.04	0.02	0.02	1.39	0.79	0.2
mouse CCR2	N/A	N/A	N/A	37.22	38.28	23.99
