# Supplementary material for: High endogenous CCL2 expression promotes the aggressive phenotype of human inflammatory breast cancer
Source: Nat Commun. 2021 Nov 25;12:6889. doi: 10.1038/s41467-021-27108-8 (PMC8617270; doi:10.1038/s41467-021-27108-8)
Supplement: Supplementary file 2 — Description of Additional Supplementary Files [file 41467_2021_27108_MOESM2_ESM.pdf]

Title: Supplementary Data 1:
Description: A3250 Canonical Pathways by IPA Analysis in vitro and in vivo. Values shown are (-log(*p*-value). *n*=3 biologically independent tumor samples, *n*=1 independent in vitro sample.

Title: Supplementary Data 2:
Description: IPA differential analysis showing both activated and inactivated pathways in A3250 compared to MDA-MB-231 tumor cells. *n*=3 independent in vitro samples per cell line. We used Fisher's exact test to determine pathways significantly enriched among DEGs. ($p < 0.05$; -log *p*-value >1.3).

Title: Supplementary Data 3:
Description: IPA differential analysis showing both activated and inactivated pathways in A3250 compared IBC3 tumor cells. *n*=3 independent in vitro samples per cell line. We used Fisher's exact test to determine pathways significantly enriched among DEGs. ($p < 0.05$; -log *p*-value >1.3).

Title: Supplementary Data 4:
Description: IPA differential analysis showing both activated and inactivated pathways in A3250 compared SUM149 tumor cells. *n*=3 independent in vitro samples per cell line. We used Fisher's exact test to determine pathways significantly enriched among DEGs. ($p < 0.05$; -log *p*-value >1.3).

Title: Supplementary Data 5:
Description: Upstream regulators activated or inhibited in A3250 compared to MDA-MB-231 tumor cells. *n*=3 independent in vitro samples per cell line. We used Fisher's exact test to determine significantly activated or inhibited upstream regulators ($p < 0.05$).

Title: Supplementary Data 6:
Description: Upstream regulators activated or inhibited in A3250 compared to IBC3 tumor cells. *n*=3 independent in vitro samples per cell line. We used Fisher's exact test to determine significantly activated or inhibited upstream regulators ($p < 0.05$).

Title: Supplementary Data 7:
Description: Upstream regulators activated or inhibited in A3250 compared to SUM149 tumor cells. *n*=3 independent in vitro samples per cell line. We used Fisher's exact test to determine significantly activated or inhibited upstream regulators ($p < 0.05$).

Title: Supplementary Data 8:
Description: Expression of human chemokines by A3250 tumors and cells in vitro determined by mRNA-Seq. Values shown are FPKM. Criteria for classifying a gene as expressed is FPKM$\geq$1 in all samples for the gene analyzed. *n*=3 biologically independent tumor samples and *n*=1 in vitro sample

in Experiment 1. *n*=3 independent in vitro samples in Experiment 2. FPKM, Fragments Per Kilobase of exon per Million mapped reads.
