# Supplementary material for: High endogenous CCL2 expression promotes the aggressive phenotype of human inflammatory breast cancer
Source: Nat Commun. 2021 Nov 25;12:6889. doi: 10.1038/s41467-021-27108-8 (PMC8617270; doi:10.1038/s41467-021-27108-8)
Supplement: Supplementary file 11 — Reporting Summary [file 41467_2021_27108_MOESM11_ESM.pdf]

nature portfolio

Corresponding author(s): Mihaela Skobe, Stuart A. Aaronson

Last updated by author(s): Oct 20, 2021

Reporting Summary

Nature Portfolio wishes to improve the reproducibility of the work that we publish. This form provides structure for consistency and transparency in reporting. For further information on Nature Portfolio policies, see our Editorial Policies and the Editorial Policy Checklist.

Statistics

For all statistical analyses, confirm that the following items are present in the figure legend, table legend, main text, or Methods section.

n/a	Confirmed	
☐	☒	The exact sample size (n) for each experimental group/condition, given as a discrete number and unit of measurement
☐	☒	A statement on whether measurements were taken from distinct samples or whether the same sample was measured repeatedly
☐	☒	The statistical test(s) used AND whether they are one- or two-sided<br>*Only common tests should be described solely by name; describe more complex techniques in the Methods section.*
☐	☒	A description of all covariates tested
☐	☒	A description of any assumptions or corrections, such as tests of normality and adjustment for multiple comparisons
☐	☒	A full description of the statistical parameters including central tendency (e.g. means) or other basic estimates (e.g. regression coefficient) AND variation (e.g. standard deviation) or associated estimates of uncertainty (e.g. confidence intervals)
☐	☒	For null hypothesis testing, the test statistic (e.g. F, t, r) with confidence intervals, effect sizes, degrees of freedom and P value noted<br>*Give P values as exact values whenever suitable.*
☒	☐	For Bayesian analysis, information on the choice of priors and Markov chain Monte Carlo settings
☐	☒	For hierarchical and complex designs, identification of the appropriate level for tests and full reporting of outcomes
☒	☐	Estimates of effect sizes (e.g. Cohen's d, Pearson's r), indicating how they were calculated

Our web collection on statistics for biologists contains articles on many of the points above.

Software and code

Policy information about availability of computer code

Data collection	No software was used for data collection.
Data analysis	BD FACSDiva software v.8.0.1 (BD Biosciences), FCS Express v.6.0 (De Novo Software), FeatureCounts package (Subread v.2.0.3), Gene Set Enrichment Analysis (GSEA) v.4.1.0, ImageJ v.1.52j, Ingenuity Pathway Analysis (IPA) Software (QIAGEN Inc. Fall Release 2021, 68752261), LivingImage software, v.4.5 (PerkinElmer), NIS Elements 3.22 (Nikon), Imaris software v.9.2 (Bitplane), RNA STAR v.2.7.9a

For manuscripts utilizing custom algorithms or software that are central to the research but not yet described in published literature, software must be made available to editors and reviewers. We strongly encourage code deposition in a community repository (e.g. GitHub). See the Nature Portfolio guidelines for submitting code & software for further information.

Data

Policy information about availability of data

All manuscripts must include a data availability statement. This statement should provide the following information, where applicable:

- Accession codes, unique identifiers, or web links for publicly available datasets
- A description of any restrictions on data availability
- For clinical datasets or third party data, please ensure that the statement adheres to our policy

The mRNA-Seq datasets generated in this study have been deposited in the Gene Expression Omnibus (GEO) database under accession code GSE158974 [https://www.ncbi.nlm.nih.gov/geo/query/acc.cgi?acc=GSE158974] and GSE180788 [https://www.ncbi.nlm.nih.gov/geo/query/acc.cgi?acc=GSE180788]. Publicly available mRNA-Seq data used in this study are available in the Gene Expression Omnibus database under accession code GSE27003 for MDA-MB-468, BT20, HCC3153 and BT474 cells [https://www.ncbi.nlm.nih.gov/geo/query/acc.cgi?acc=GSE27003] and GSM3145605 for MDA-IBC3 [https://www.ncbi.nlm.nih.gov/geo/query/acc.cgi?acc=GSM3145605]. Microarray datasets for the human IBC transcriptomic analysis conducted in this study are available in the EBI ArrayExpress database under

accession code E-MTAB-1006 [https://www.ebi.ac.uk/arrayexpress/experiments/E-MTAB-1006/] as well as the GEO database under accession code GSE22597 [https://www.ncbi.nlm.nih.gov/geo/query/acc.cgi?acc=GSE22597]. Remaining data are available within the Article, in Supplementary Data or Tables, and in the Source Data file. There are no restrictions on data availability.

Field-specific reporting

Please select the one below that is the best fit for your research. If you are not sure, read the appropriate sections before making your selection.

- [x] Life sciences
- [ ] Behavioural & social sciences
- [ ] Ecological, evolutionary & environmental sciences

For a reference copy of the document with all sections, see nature.com/documents/nr-reporting-summary-flat.pdf

Life sciences study design

All studies must disclose on these points even when the disclosure is negative.

Sample size	qPCR sample sizes were determined based on previously published power analyses on low-abundant gene targets (doi: 10.1128/AEM.69.11.6597-6604.2003). Sample sizes for cell culture and mRNA-seq experiments were selected to be at least n=3 based on standards in the literature. Sample sizes for in vivo tumor and metastasis studies were determined based on pilot studies in mice, which established tumor and metastasis incidence, and variability in data between the individual mice.
Data exclusions	No data were excluded from analyses.
Replication	mRNA-Seq experiments and qPCR analyses were performed twice. Cell culture experiments were repeated at least twice. Primary tumor experiments were repeated 4x with different investigators involved, for tissue and data collection. Metastasis experiments (Fluc measurements) were repeated twice. FACS of tumors was repeated twice. All attempts at replication were successful for the aforementioned experiments.
Randomization	Age-matched mice were randomized for tumor injection.
Blinding	Investigators were blinded to group allocation during Fluc data collection and analysis of tumors and metastases in mouse experiments. Investigators were blinded for analyses of tissues by immunostaining and histology, and also for data collection by FACS.

Reporting for specific materials, systems and methods

We require information from authors about some types of materials, experimental systems and methods used in many studies. Here, indicate whether each material, system or method listed is relevant to your study. If you are not sure if a list item applies to your research, read the appropriate section before selecting a response.

Materials & experimental systems

n/a	Involved in the study	
[ ]	[x]	Antibodies
[ ]	[x]	Eukaryotic cell lines
[x]	[ ]	Palaeontology and archaeology
[ ]	[x]	Animals and other organisms
[x]	[ ]	Human research participants
[x]	[ ]	Clinical data
[x]	[ ]	Dual use research of concern

Methods

n/a	Involved in the study	
[x]	[ ]	ChIP-seq
[ ]	[x]	Flow cytometry
[x]	[ ]	MRI-based neuroimaging

Antibodies

Antibodies used

Immunofluorescent staining:
Alexa Fluor 488 F(ab')2-goat anti-rabbit IgG, Invitrogen #A-11070 (1:300)
Alexa Fluor 555 goat anti-rat IgG, Invitrogen #A-21434 (1:300)
Alexa Fluor 488 goat anti-rabbit IgG, Invitrogen #A-11037 (1:300)
Anti-GFP, Abcam ab6556 (1:100)
Anti-mouse CD11b clone M1/70, Biolegend 101201 (1:200)
Anti-mouse CD34 clone RAM34, Invitrogen 14-0341-82 (1:100)
Anti-mouse CD45, Abcam ab10558 (1:100)
Anti-mouse F4/80 clone A3-1, Bio-Rad MCA497 (1:100)
Anti-mouse Ly-6G clone 1A8, Biolegend 127601 (1:100)
Anti-mouse LYVE-1, Abcam ab14917 (1:600)
DAPI, Sigma-Aldrich D9542 (10 µg/ml)

Immunohistochemistry:

Anti-mouse CD45, Abcam ab10558 (1:1000)
Anti-human ErbB2 [EP1045Y], Abcam ab134182 (1:100)
Anti-Estrogen receptor-alpha, BioGenex NU710-UC (1:50)
Anti Progesterone receptor [SP2], Abcam ab16661 (1:400)
Anti-mouse Iba1, Wako 019-19741 (1:1000)
Anti-mouse Ki-67, Leica Biosystems NCL-Ki67p (1:1000)
Wide-spectrum cytokeratin, Abcam ab9377 (1:100)
Anti-human CD68, Dako M0814 (1:100)
Anti-human CD66b, BD Pharmingen (BD Biosciences) 555723 (1:600)

Flow cytometry:
Anti-mouse CCR2-BV605 clone SA203G11, Biolegend 150615 (1μg/ml)
Anti-mouse CD11b-APC clone M1/70, Biolegend 101212 (1μg/ml)
Anti-mouse CD45-BV785 clone 30-F11, Biolegend 103149 (1μg/ml)
Anti-mouse F4/80-FITC clone BM8, Biolegend 123108 (1μg/ml)
Anti-mouse Ly6C-BV421 clone HK1.4, Biolegend 128032 (1μg/ml)
Anti-mouse Ly6G-PE clone 1A8, Biolegend 127608 (1μg/ml)

Western Blot:
Alexa Fluor 680 goat anti-mouse IgG, Invitrogen A-21059 (1:10000)
Anti-human CCL2/JE/MCP-1 clone 23007, R&D Systems MAB679 (1μg/ml)

Validation

Primary antibodies were validated for species specificity and applications by the manufacturers, as stated on their websites:

Anti-GFP - Reactive against all variants of Aequorea victoria GFP such as S65T-GFP, RS-GFP, YFP, CFP, RFP and EGFP. Validated for electron Microscopy, IHC-P, IHC-Fr, WB, ICC/IF

Anti-mouse CD11b clone M1/70 - Validated for immunocytochemistry (ICC) and frozen immunohistochemistry (IHC-F) in mouse

Anti-mouse CD34 clone RAM34 - Validated by Flow Cyt analysis of mouse bone marrow cells

Anti-mouse CD45 - Validated in WB, IHC, Flow Cyt and tested in mouse, rat, human

Anti-mouse F4/80 clone A3-1 - recognizes murine F4/80. Validated in Flow Cyt, IF, Immuno-electron microscopy, IHC, IP, Radioimmunoassays, and WB

Anti-mouse Ly-6G clone 1A8 - Validated for Flow Cyt and IHC-F in mouse

Anti-mouse LYVE-1 - Validated in IHC and validated in mouse

Anti-mouse CD45 - Validated in WB, IHC, Flow Cyt and validated in mouse, rat, human

Anti-human ErbB2 [EP1045Y] - Validated in WB, IP, IHC, ICC/IF and validated in human

Anti-Estrogen receptor-alpha - Validated in IHC in human

Anti Progesterone receptor [SP2] - Validated in IHC, ICC/IF, Flow Cyt in human

Anti-mouse Iba1 - Validated for immunohistochemistry (IHC) and immunocytochemistry in mouse, rat, human

Anti-mouse Ki-67 - Validated for immunohistochemistry on paraffin sections in mouse

Wide-spectrum cytokeratin - Validated in IHC and tested in mouse, cow, dog, human

Anti-human CD68 - Validated for immunohistochemistry (IHC) in human

Anti-human CD66b - Validated for flow cytometry and fluorescence microscopy in human

Anti-mouse CCR2-BV605 clone SA203G11 - Validated for flow cytometry in mouse

Anti-mouse CD11b-APC clone M1/70 - Validated for flow cytometry in mouse

Anti-mouse CD45-BV785 clone 30-F11 - Validated for flow cytometry in mouse

Anti-mouse F4/80-FITC clone BM8 - Validated for flow cytometry in mouse

Anti-mouse Ly6C-BV421 clone HK1.4 - Validated for flow cytometry in mouse

Anti-mouse Ly6G-PE clone 1A8 - Validated for flow cytometry in mouse

Anti-human CCL2/JE/MCP-1 clone 23007 - Validated for WB, IHC, ELISA and neutralization in human

BD Biosciences tests its antibodies on "primary cells, cell lines and/or transfectant cell models with relevant controls using multiple immunoassays to ensure biological accuracy."
Abcam antibodies are knock-out validated or validated in cells with established levels of protein expression. Antibodies used in IF are validated by "looking at cells that either do or do not express the target protein within the same tissue."
Invitrogen and Sigma use cell treatment antibody validation methods where antibodies directed against pan proteins are tested in multiple cell lines that express varying levels of protein across different species.
Biolegend antibodies are produced from hybridomas and tested for validity through ELISA or application-specific screening of antibodies from clones and subsequent application testing.
Bio-Rad employs immunocapture followed by mass spectrometry analysis to identify proteins that interact directly with the purified antibody, as well as additional proteins that interact indirectly with the target protein.
R&D Systems "antibodies are tested for cross-reactivity with closely related molecules using a variety of applications, including direct ELISA, to ensure specificity."
BioGenex validates their primary antibodies through ELISA testing.
Dako (Agilent) concentrate antibody and Wako (Fujifilm) antibodies are validated through cross-lot testing and on end user operating procedures.
Leica do not provide information on validation methods on their web site.

Eukaryotic cell lines

Policy information about cell lines

Cell line source(s)	The A3250 breast carcinoma cells were isolated from a primary tumor of a 50-year old de-identified patient with IBC. IBC tumor was obtained from Dr. Dirk Iglehart, Duke University, in 1985.<br>We generated A3250 cells expressing eGFP and firefly luciferase, and the A3250-shCtrl and A3250-shCCL2 cell lines.<br>The SUM149 IBC cell line was obtained from Dr. Stephen Ethier (Medical University South Carolina). The HCC3153 cell line was obtained from Dr. John Minna, UT Southwestern.<br>MDA-MB-157, MDA-MB-231, SKBR3 and MCF7 cell lines were obtained from ATCC, and we generated the MDA-MB-231-eGFP-Fluc cell line.
Authentication	The A3250 cell line was authenticated by STR analysis at the Department of Oncological Sciences DNA sequencing core, Mount Sinai. SUM149, MDA-MB-157, MDA-MB-231, HCC3153, SKBR3, and MCF7 cell lines were also authenticated using STR analysis.
Mycoplasma contamination	All cell lines tested negative for mycoplasma contamination by qPCR.
Commonly misidentified lines (See ICLAC register)	No commonly misidentified cell lines were used in this study.

Animals and other organisms

Policy information about studies involving animals; ARRIVE guidelines recommended for reporting animal research

Laboratory animals	Eight to nine-week old female SCID/NCr (Strain 561) mice were used in most mouse experiments. Mice were ether purchased from Charles River Laboratories or bred in-house. Seven-week old female NOD SCID (Strain 394) mice were used in one experiment (Strain 394), from Charles River Laboratories. Animals were housed at Mount Sinai's Center for Comparative Medicine and Surgery in climate-controlled barrier facilities with automated light/dark cycles.
Wild animals	The study did not involve wild animals.
Field-collected samples	The study did not involve samples collected from the field.
Ethics oversight	Mouse experiments were performed in accordance with protocols approved by the Icahn School of Medicine at Mount Sinai's Institutional Animal Care and Use Committee (IACUC) according to federal and institutional guidelines.

Note that full information on the approval of the study protocol must also be provided in the manuscript.

Flow Cytometry

Plots

Confirm that:

☒ The axis labels state the marker and fluorochrome used (e.g. CD4-FITC).

☒ The axis scales are clearly visible. Include numbers along axes only for bottom left plot of group (a 'group' is an analysis of identical markers).

☒ All plots are contour plots with outliers or pseudocolor plots.

☒ A numerical value for number of cells or percentage (with statistics) is provided.

Methodology

Sample preparation	Xenograft mouse tumors ~5 mm in diameter, spleens and lungs were minced into 1 mm^3 pieces. Tissues were digested in collagenase D (Sigma 11088858001) using the MACSmix tube rotator (Miltenyi) at 37°C, and pressed through a 70 µm filter. Lymph nodes were dissociated by mechanical disruption. Blood was collected by cardiac puncture. Erythrocytes in blood, spleen and lung were lysed using RBC lysis buffer (eBioscience 00-4300-54), as per the manufacturer's protocol. Cells were counted using the Countess II Automated Cell Counter (Invitrogen). Samples were stained with antibodies on ice for 30 minutes in FBS Stain Buffer (BD Pharmigen 554656), and run on the BD LSRFortessa X-20 cell analyzer.
Instrument	BD LSRFortessa X-20 cell analyzer
Software	BD FACSDiva software v.8.0.1 (BD Biosciences), FCS Express version 6.0 (De Novo Software)
Cell population abundance	Sorting was not performed.
Gating strategy	Gating strategy is illustrated in Figure 5 and in Supplemental Figure 5b. Cells were identified on an FSC-A/SSC-A plot and doublets were excluded using an FSC-A/FSC-H plot. Viable cells were identified using FSC-A/DAPI plot. CD45+ cells were identified on FSC-A/CD45 plot. CD11B+ myeloid cells were identified on CD45/CD11B plot. Boundaries between positive and negative staining cell populations were defined using Fluorescence Minus One controls.

☒ Tick this box to confirm that a figure exemplifying the gating strategy is provided in the Supplementary Information.
